# A framework for stability‐based module detection in correlation graphs

**DOI:** 10.1002/sam.11495

**Published:** 2021-01-08

**Authors:** Mingmei Tian, Rachael Hageman Blair, Lina Mu, Matthew Bonner, Richard Browne, Han Yu

**Affiliations:** ^1^ Department of Biostatistics State University of New York at Buffalo Buffalo New York USA; ^2^ Department of Epidemiology and Environmental Health State University of New York at Buffalo Buffalo New York USA; ^3^ Department of Biotechnical and Clinical Laboratory Sciences State University of New York at Buffalo Buffalo New York USA; ^4^ Department of Biostatistics and Bioinformatics Roswell Park Comprehensive Cancer Center Buffalo New York USA

**Keywords:** clustering, graphical model, Jaccard coefficient, module detection, network, stability

## Abstract

Graphs can be used to represent the direct and indirect relationships between variables, and elucidate complex relationships and interdependencies. Detecting structure within a graph is a challenging problem. This problem is studied over a range of fields and is sometimes termed community detection, module detection, or graph partitioning. A popular class of algorithms for module detection relies on optimizing a function of modularity to identify the structure. In practice, graphs are often learned from the data, and thus prone to uncertainty. In these settings, the uncertainty of the network structure can become exaggerated by giving unreliable estimates of the module structure. In this work, we begin to address this challenge through the use of a nonparametric bootstrap approach to assessing the *stability* of module detection in a graph. Estimates of stability are presented at the level of the individual node, the inferred modules, and as an overall measure of performance for module detection in a given graph. Furthermore, bootstrap stability estimates are derived for complexity parameter selection that ultimately defines a graph from data in a way that optimizes stability. This approach is utilized in connection with correlation graphs but is generalizable to other graphs that are defined through the use of dissimilarity measures. We demonstrate our approach using a broad range of simulations and on a metabolomics dataset from the Beijing Olympics Air Pollution study. These approaches are implemented using bootcluster package that is available in the R programming language.

## 1 INTRODUCTION

Networks have been used widely to graphically depict complex relationships between entities in biological and social systems. Modules in a network arise naturally when nodes exhibit a high degree of connectivity to each other, and a lower degree of connectivity to others [11, 29]. Network structure of this type is referred to slightly differently across the various fields that utilize it, for example, sub‐group, community, and module. Over the past 50 years, several module detection algorithms have been proposed [7, 16]. A unifying underlying assumption to module detection approaches is that the network structure, which is represented by a binary adjacency matrix, is known exactly. However, in many applications, the network structure is learned from data and thus prone to uncertainty. This uncertainty can propagate and give rise to unreliable estimations of module structure.

Module detection shares many of the same fundamental challenges as the more general clustering problem, as they both aim to identify groups (i.e., clusters or modules) of similar items from data. In fact, the problem of module detection in a graph is often referred to as graph partitioning or graph clustering. The unsupervised nature of these problems poses challenges for the assessment of the performance of a method, the estimation of reproducibility or quality of individual groups, and also for the selection of the number of groups. In lieu of a gold standard, different forms of cluster *stability* have been used as a surrogate to assess performance. Stability estimates capture how stable the clusterings are over several different representations of the data, which are derived through subsetting, cross‐validation, data noising or re‐sampling, among others [4, 12, 14, 15, 20, 22, 26, 36, 37, 38].

Meinshausen and Bühlmann [27] developed an approach to estimating stability in a graph. The primary objective centered on utilizing a form of stability parameter estimation that controls the graph structure, as it applies to structural learning of a graphical model. This approach to stability leverages sub‐sampling in connection with a high‐dimensional selection algorithm (lasso), to identify a parameter that gives rise to a stable graph. This method was later extended to Gaussian graphical models for the purpose of selection and improvement of gene expression signatures' stability [10, 18]. Notably, these approaches are restricted to the identification of stable graphs.

Recently, Yu et al. [38] developed an approach to cluster stability estimation. As with other bootstrap approaches to stability, an overall estimation of stability can provide an assessment of cluster quality [12, 14]. A distinguishing feature of this method is that stability can be assessed at the level of the item being clustered, the clusters themselves, and can also be used for cluster parameter selection. The interpretation of stability in these contexts facilitates improved interpretation of the clustering results, thereby allowing us to quantify uncertainty across these levels.

In this work, the cluster stability framework of Yu et al. [38] is extended to the problem of module detection in a graph. This approach to stability utilizes the bootstrap [13] for data re‐sampling, construction of a graph, and subsequent module detection. For each bootstrap replication, we compare the compositions of the modules to the original modules detected from the data. This comparison is made across node pairs to avoid the need for module matching between comparisons. The output is an estimation of stability that is based on a Jaccard index and computed at the level of the individual node membership to the module, the detected modules, and an overall measure of the graph clustering performance.

The major contribution of this paper is a novel *stability* framework that characterizes modules in a graph. This flexible framework can be used to quantify stability at the level of the individual node, the individual modules, as well as an overall stability assessment of the method and parameterizations. This approach can also be used to identify the optimal number of modules, that is, model selection, in a graph, a longstanding challenge in the community/module detection field, and more broadly in the area of data clustering. To the best of authors' knowledge, this represents the first approach to define module stability in a graph and the first use of this measure for model selection. This novel approach is benchmarked on several simulations that examine performance as a function of the number of parameters, sample size, correlation across inputs, and the number of modules. This framework is also applied to a high‐dimensional air pollution exposure metabolomics dataset collected during the Beijing Olympics [28]. The module stability framework has been implemented in the open‐source R package bootcluster available on CRAN.

## 2 MATERIALS AND METHODS

**FIGURE 1:**
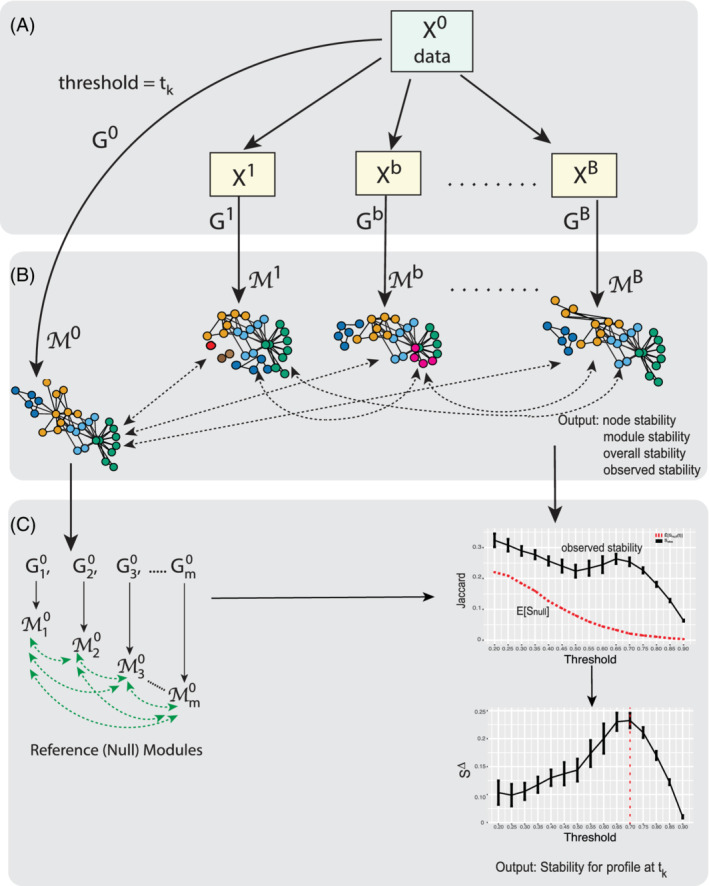
A schematic of the workflow for bootstrapping module stability. (A) A graph, *G* ^0^ is derived from *X* ^0^ based on correlation. The observations in the data, *X* ^0^, are bootstrapped to produce {*X* ^1^, *X* ^2^, …, *X* ^*B*^} and corresponding graphs {*G* ^1^, *G* ^2^, …, *G* ^*B*^} are formed based on correlation. (B) A module detection algorithm is applied to *G* ^0^ and each of the graphs derived from the bootstrap samples. Pairwise memberships of nodes within modules are compared between the modules arising, {*M*^0^, *M*^1^, …, *M*^*B*^}, from the bootstrapped graphs. The output is stability estimates at the level of the individual node, the module, overall, and a measure of observed stability. (C) Random graphs assuming no module structures were generated by conserving the degree sequence in the original graph. Changes in node memberships between module sets are quantified via Jaccard coefficients, and used to derive the expected null stability, *E*[*S*_null_]. These are included in the output stability profile, *S*^Δ^ = *S*^obs^ − *E*[*S*_null_], at the threshold *t*_*k*_

In this section, we will first describe a framework for the estimation of module stability in networks and its utilization in selecting the cut‐off for constructing the correlation network. In this framework, we defined the stability (1) at the level of the individual nodes being clustered into modules, (2) for the modules themselves, (3) as an overall measure (as a surrogate for performance) of the module detection, and (4) for model selection. Stability calculations are obtained through repeated re‐sampling and subsequent network and community detection using the nonparametric bootstrap [13]. The calculations rely on the preservation of pairwise memberships of nodes to a module, between the original data, and bootstrapped replicates. We will describe the algorithm from the foundation of the correlation graph to the module detection, the stability estimates, and finally to its application to parameter (model) selection for correlation network construction. A schematic depicting the work‐flow for this method is presented in Figure 1. The stability estimation procedure described below is fully detailed in Algorithm 1.

*Correlation graphs*: Let *X* ^0^ ∈ **R** ^*N*×*p*^ denote the data matrix with *N* observations and *p* variables. Without loss of generality, suppose we want to identify network modules from an undirected graph, *G* ^0^, that is defined from the correlation matrix *C* ^0^ ∈ **R** ^*p*×*p*^. Specifically, suppose that the graph, *G* ^0^, is represented by a binary adjacency matrix, with edges between nodes *n*_*i*_ and *n*_*j*_ exist if and only if ∣*C* ^0^(*i*, *j*)∣ > *t*, where *t* is a constant threshold for the absolute correlation. Our method for stability estimation relies *B* bootstrap replications of the dataset, *X* ^1^, *X* ^2^, …, *X* ^*B*^, where the *N* observations are re‐sampled with replacement. For each bootstrapped dataset, a corresponding correlation matrix is formed, *C* ^1^, *C* ^2^, …, *C* ^*B*^, and a graph, *G* ^1^, *G* ^2^, …, *G* ^*B*^ is created based on the same threshold, *t*, that was used on the original data (Figure 1A).

*Module detection*: The module detection that is implemented in this work relies on a fast greedy algorithm that utilizes a form of greedy optimization of modularity [30]. Briefly, we describe the approach and refer the reader to [30] for additional details. Without loss of generality, suppose that there are *U* modules in a particular network. In mathematical terms, we define *E* ∈ **R** ^*U*×*U*^ to be a symmetric matrix, with *e*_*ij*_ defined as the proportion of edges in the network that connects nodes in module *i* to those in module *j*. The fraction of edges in the network that connects the nodes in the same module is given by the trace of this matrix, $\mathrm{Tr}\left(E\right)=\sum_{i}e_{\mathrm{ii}}$. The fraction of edges that connect to nodes in module *i* can be defined as the row (or column) sums, $s_{i}=\sum_{j}e_{\mathrm{ij}}$. Notably, the relationship *e*_*ij*_ = *s*_*i*_ · *s*_*j*_ would hold in a network that contains random edges between nodes in the network, without any regard for module structure. The modularity function that is maximized in the fast greedy algorithm is defined as the difference between the proportion of edges within a module, and those that are expected to occur at random without the consideration of community structure:

(1) $$Q=\sum_{i}\left(e_{\mathrm{ii}}-s_{i}^{2}\right).$$

Note that the interpretation of *Q* ≤ 0 suggests no structure or as much structure as would be expected by chance. On the other hand, *Q* > 0 (in practice, *Q* > 0.3) suggests strong structure [30]. The optimization of modularity is performed using an agglomerative clustering approach that produces a dendrogram containing nodes in the network. The dendrogram is then cut to produce modules that maximize Equation (1). Clauset et al. [6] improved the runtime for this optimization problem, making it scalable to very large networks (e.g., 400,000 nodes and 2 million edges) [30]. This approach is implemented in the igraph package [8].

*Bootstrap procedure*: Stability estimation utilizes bootstrapping to estimate the stability of clusterings [38]. However, in our setting, the nodes in a network are variables instead of subjects, therefore are typically fixed during the sampling process. The randomness of the sampling process comes from the random subjects drawn from the population, and is reflected by the sampling distribution of the correlation coefficients between each pair of variables, or the overall correlation matrix. Therefore, the correct resampling scheme is to randomly draw *N* subjects with replacement, which is a key difference from Yu et al. [38]. This resampling will be repeated *B* times such that we will have *B* resampled graphs *G*^1^, …, *G*^*B*^. We define, ℳ^0^, as the set of modules corresponding to the reference graph, *G* ^0^, and ℳ^1^, …, ℳ^*B*^ as the sets of modules corresponding to *B* graphs from the bootstrapped data (Figure 1B). Note that the numbers of modules in the reference graph and bootstrapped graphs do not need to be the same.

*Stability estimates*: The approach to stability estimation is to compare the module composition of the reference correlation graph to the various bootstrapped correlation graphs, and to assess the stability at the (1) node‐level, (2) module‐level, and (3) overall. Following Yu et al. [38], we define stability at these three levels. The approach relies on the use of Jaccard coefficient to quantify changes in pairwise co‐membership of nodes between two sets of modules ℳ^0^ and ℳ^*b*^. Specifically, the similarity of two modules with respect to a node *n*_*i*_ is defined as:

$$A\left(n_{i},{ℳ}^{0},{ℳ}^{b}\right)≔\text{Jaccard}\left({ℳ}^{0}\left(n_{i}\right),{ℳ}^{b}\left(n_{i}\right)\right),$$

where ℳ^0^(*n*_*i*_) and ℳ^*b*^(*n*_*i*_) are the modules containing *n*_*i*_ in ℳ^0^(*n*_*i*_) and ℳ^*b*^(*n*_*i*_), respectively. This definition is based on an approximation of the Hamming distance between partitions and its decomposition into observation‐wise quantities, see [38] for details. The overall similarity is defined as the expectation of observation‐wise similarity, thus the sample overall similarity can be obtained as:

(2) $$A\left({ℳ}^{0},{ℳ}^{b}\right)=\frac{1}{p}\sum_{i=1}^{p}A\left(n_{i},{ℳ}^{0},{ℳ}^{b}\right).$$

Let {ℳ^0^, ℳ^1^, …, ℳ^*B*^} be the sets of modules detected from original graph, *G* ^0^, and the graphs from the *B* re‐sampled datasets, {*G* ^1^, …, *G* ^*B*^}. The node and module stability are defined as follows:

(3) $$S^{\text{node}}\left(n_{k}|{ℳ}^{0},G^{0}\right)=\frac{1}{B}\sum_{j=1}^{B}A\left(n_{k},{ℳ}^{0},{ℳ}^{j}\right),$$

(4) $$\begin{array}{cc} S^{\text{module}}\left(M_{i}^{0}|{ℳ}^{0},G^{0}\right) & =\frac{1}{\left|M_{i}^{0}\right|}\sum_{n_{k}\in M_{i}^{0}}\sum_{j=1}^{B}A\left(n_{k},{ℳ}^{0},{ℳ}^{j}\right)\\ & =\frac{1}{\left|M_{i}^{0}\right|}\sum_{n_{k}\in M_{i}^{0}}S^{\text{node}}\left(n_{k},{ℳ}^{0}\right). \end{array}$$

The overall stability estimate is defined as:

(5) $$S^{\text{over}}\left({ℳ}^{0}|G^{0}\right)=\frac{1}{B}\sum_{j=1}^{B}A\left({ℳ}^{0},{ℳ}^{j}\right).$$

Note that the estimate of stability in Equation (5) is conditional on the modules, ℳ^0^, detected from the original data, *X* ^0^, and graph, *G* ^0^.

*Threshold selection*: The binary adjacency matrix for a graph is constructed from a correlation matrix based on a predetermined threshold *t*. This approach of module stability estimation can be extended to the problem of the selection of an optimal threshold, *t*_opt_, that produces the most stable graph and subsequent modules. The key assumption is that the nodes in the underlying correlation network are organized in module structures, so we seek to estimate the true threshold *t* so as to yield a network with the most stable module memberships for the nodes. However, it is notable that the stability is not directly comparable among networks with different edge densities. An extreme case is a fully connected network *t* = 0, which will be perfectly stable since all nodes will always be in the same module that covers the entire network. The other extreme is an edgeless graph *t* > 1, where all nodes will always be their own module, thus will also be stable. With such difference untended, we will always select the trivial solutions based on maximum stability.

Therefore, in an effort to assess the quality of the module structure, we take an approach that is similar to the spirit of the gap statistic for the selection of the number of clusters [35]. For each threshold, *t*, that is considered, we standardize the observed stability *S* ^obs^, named as *S* ^Δ^, by comparing it against the expectation under a null reference graph that contains no module structure that is, *E*[*S*_null_]. We also define an unconditional estimate of the stability, which compares in a pair‐wise manner, the sets of modules estimated between all of the bootstrap replications:

(6) $$S^{\mathrm{obs}}=\frac{1}{B\left(B+1\right)}\sum_{i=0}^{B}\sum_{j=0,j\neq i}^{B}A\left({ℳ}^{i},{ℳ}^{j}\right)=\frac{1}{B+1}\sum_{i=0}^{B}S^{\text{over}}\left({ℳ}^{i}\right).$$

Note that this definition of unconditional stability cannot be defined at the module or node levels. Therefore, it is not particularly useful in the context of the overall interpretation of the individual node and module stabilities. Rather, Equation (6) can be utilized for the selection of an optimal threshold, *t*, as it provides a measure of overall stability of the data and method.

Finally, we define the overall Jaccard stability as:

(7) $$S^{\Delta }\left(t\right)=S^{\mathrm{obs}}\left(t\right)-E\left[S^{\text{null}}\left(t\right)\right],$$

where the unconditional observed stability *S* ^obs^ is estimated as in Equation (6). The expected stability is derived from a simulated ensemble of reference graphs, denoted as $G_{1}^{0},\ldots ,G_{m}^{0}$. The random graphs preserve the degree and node ordering of the reference graph, *G* ^0^, but no module structures are assumed to be present [9]. The expected stability under the null is estimated by the overall stability among the random graphs. The modules detected in each of the *B* bootstrap replications are compared to the estimated stability under the null. Specifically, comparisons are made between modules, ${ℳ}_{1}^{0},\ldots ,{ℳ}_{m}^{0}$, from the full set of random graphs, $G_{1}^{0},\ldots ,G_{m}^{0}$. We define the expectation as:

(8) $$\hat{E}\left[S_{\text{null}}\left(t\right)\right]=\frac{1}{m\left(m-1\right)}\sum_{i=1}^{m}\sum_{i\neq j}^{m}A_{r}\left({ℳ}_{i}^{0},{ℳ}_{j}^{0}\right),$$

where $A_{r}\left({ℳ}_{i}^{0}{ℳ}_{j}^{0}\right)$ is the agreement between two module partitions ${ℳ}_{i}^{0}$ and ${ℳ}_{j}^{0}$ calculated using Equation (2). This process is depicted in Figure 1C.

Algorithm 1 **Bootstrapping module stability in a correlation network**

**Initialize using the full data**:

**Input:** *X* ^0^ ∈ **R** ^*N*×*p*^, *t* = {*t*_1_, …, *t*_*k*_} ∈ **R**^*k*^, *C*_0_ ∈ **R** ^*p*×*p*^

**for** *l* = 1 to *k* **do**

1. Construct *G* ^0^ ∈ **R** ^*p*×*p*^ with edges *G* ^0^(*i*, *j*) = 1 if $\left|c_{i,j}^{0}\right|>t_{l}$.

2. Apply module detection algorithm to *G* ^0^ obtain ℳ^0^.

3. Generate random graphs, $G_{1}^{0},\ldots ,G_{m}^{0}$, from *G* ^0^.

**Output for each threshold: *G*** ^**0**^, ℳ^0^, $G_{1}^{0},\ldots ,G_{m}^{0}$

**end for**

**Bootstrap stability estimation**:

**Input:** *t*_*l*_, *X* ^0^, *G* ^0^, ℳ^0^, $G_{1}^{0},\ldots ,G_{m}^{0}$

**for** *b* = 1 to *B* **do**

1. Generate a bootstrap sample *X* ^*b*^ ∈ **R** ^*N*×*p*^

2. Calculate correlation matrix for *C* ^*b*^ ∈ **R** ^*p*×*p*^

3. Create graphs, *G* ^*b*^, with edges *G* ^*b*^(*i*, *j*) = 1 between nodes if $\left|c_{i,j}^{b}\right|>t_{l}$

4. Apply module detection algorithm to *G* ^*b*^ to obtain ℳ^*b*^

5. Calculate $A\left({ℳ}^{0}{ℳ}^{b}\right)=\frac{1}{p}\sum_{i=1}^{p}A\left(n_{i}{ℳ}^{0}{ℳ}^{b}\right)$

**end for**

**Output**:

Calculate *S*^node^(*n*_*k*_|ℳ^0^, *G*^0^) using Equation (3)

Calculate $S^{\text{module}}\left(M_{i}^{0}|{ℳ}^{0}G^{0}\right)$ for all $M_{i}^{0}\in {ℳ}^{0}$ using Equation (4)

Calculate *S*^over^(ℳ^0^|*G*^0^) using Equation (5)

Calculate the stability profile using Equation (7) for all values of *t*.

Select *t* so as to maximize *S* ^Δ^.

### 2.1 Simulations

The first set of simulations were performed for a broad range of scenarios using a block diagonal correlation graph. This structure enabled us to investigate the performance of the stability approach under different strengths, varying sample sizes, and graph sizes. The second set of simulations were carried out to examine performance on an immunoglobulin interaction network [17]. The two simulation frameworks are detailed below.

*Simulation 1*: *Block diagonal model*: Module structure can be represented as a binary block diagonal matrix, where the number of the main‐diagonal blocks is equal to the number of modules in the graph. The basis of our simulations relies on a correlation graph that is generated from a given block diagonal matrix. A value of 1 is assigned to the diagonal elements, and we define the constants *α* and *β* that are assigned to the main‐diagonal blocks and off‐diagonal blocks, respectively. We also define a threshold value *t*, such that *β* ≤ *t* < *α*, which enables us to transform a correlation matrix to an adjacency matrix, and subsequent graph.

This framework enables us to obtain the *true modules* based on this predetermined adjacency matrix, which will be used to assess performance in model selection for the optimal threshold, *t*_opt_. The adjusted rand index (ARI) was calculated between the detected modules with the selected threshold and the true modules. Our method was compared against a traditional approach that utilizes *p*‐values from the pairwise correlation to construct the graph. The psych package was used to perform the correlation analysis, which conveniently calculates the correlation coefficients and the corresponding *p*‐values. A Bonferroni adjustment was made to account for multiple testing. An edge was placed between nodes if the adjusted *p*‐value was less than 0.05 [2, 34].

Data were simulated for a range of different correlation matrices where *β* = 0.1 and *α* ∈ {0.3, 0.4, 0.5, 0.6, 0.7, 0.8}, respectively. Under this block‐diagonal model, we also set out to investigate the stability framework under different strengths of association (*block*), numbers of nodes (*p*), sample sizes (*N*), and numbers of modules. Data were simulated while ranging two of these parameters at a time, as described below. For each simulated dataset, we calculated the corresponding overall Jaccard stability using Equation (7) across a range of thresholds from 0.3 to 0.9.

1. Varying the number of observations, *N* ∈ {30, 50, 100}, and the strength of association (*α* = block values), *α* ∈ {0.3, 0.4, 0.5, 0.6, 0.7, 0.8}, simultaneously. The number of variables is fixed at 100, and the number of modules is fixed at 10 for all thresholds, *t*, considered.
2. Varying the number of nodes (variables), *p* ∈ {60, 100, 200}, and the block values, *α* ∈ {0.3, 0.4, 0.5, 0.6, 0.7, 0.8}, simultaneously. The number of observations is fixed at *N* = 100, and the number of modules is fixed at 10 for all thresholds, *t*, considered.
3. Varying the number of modules, modules ∈ {2, 4, 6, 8, 10}, and the number of nodes (variables), *p* ∈ {60, 100, 200}, simultaneously. The number of observation is fixed at 100, and the strength of association (block values) is fixed at 0.5 for all threshold, *t*, considered.

*Simulation 2*: *Immunoglobulin interaction graph*: Graphs based on an immunoglobulin (immuno) interaction graphs [17] were simulated using the igraphdata package. Briefly, the immuno graph has 1316 nodes that represent amino acids. Our simulations were based on a subgraph that was generated from the detected modules. As a first step, we identified seven modules in the immuno data using the fast greedy algorithm [30]. Second, we captured each modules information in terms of nodes and then constructed a series of nested subgraphs from these modules in order to generate the graphical model, that is, the number of modules ∈ {2, 3, 4, 5, 6, 7}, respectively. Third, the undirected Gaussian graphical Markov models (GMMs) [1] based on each subgraph was simulated using the R package qpgraph package [5]. Finally, we generated *N* = 30, 50, 100 random samples from the multivariate normal distribution, which is defined by the class of undirected Gaussian GMMs. Taken together, there were a total of 18 different nested graphs considered in this simulation.

### 2.2 Applications to air pollution metabolomics data

The approach to bootstrapping stability was also applied to a high‐throughput metabolomics dataset. The data were collected from 26 participants at three‐time points, which were before, during and after, the 2008 Beijing Olympics [28]. During the Olympics, there were government‐mandated restrictions that aimed to reduce pollution levels and thus decreased exposure. The data consists of a total of *p* = 886 metabolites (nodes) measured by Metabolon using mass spectrometry, see [28] for data processing details. Eight metabolites were eliminated from the analysis based on severe missing data (<50%). Our objective was to identify significant concerted changes in metabolites through the use of module detection. In this application, we applied Algorithm 1 to metabolite correlation networks with a sequence of *t* from 0.2 to 0.9 at a step of 0.05.

## 3 RESULTS

Our approach to estimating module stability has several objectives: to identify a threshold for defining a correlation graph that gives rise to stable and reproducible modules, to characterize the stability of individual modules, and to characterize the stability of the membership of nodes to a given module. The building blocks of these different stability measures are based on the pairwise changes in the membership of node pairs between modules detected in the original data, and modules detected from bootstrap replications of the data.

**FIGURE 2:**
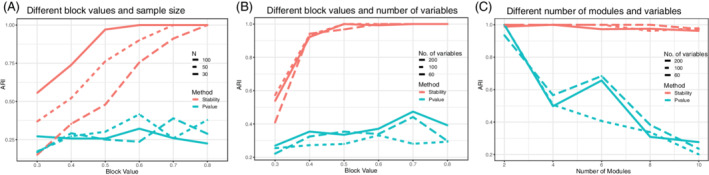
Adjusted rand index (ARI) for three sets of different simulations in Simulation 1. The bootstrapped stability (red) is shown to be consistently better than a *p*‐value approach (blue). Simulation settings are as follows. (A) Varying the sample size and block value, which reflects the strength of association of the modules. (B) Varying the parameters and block values. (C) Varying the number of parameters and the number of communities

Our first set of simulations utilized a block diagonal model to generate the data with varying sample sizes, number of variables (i.e., network sizes), and the number and strengths of modules. Within these simulation settings, we compared our approach to a standard approach that is based on the correlation of *p*‐values to derive the graph, followed by module detection. Performance was compared using ARI for different sample sizes and strengths of association (Figure 2A), different numbers of nodes (variables) and strengths of association (Figure 2B) and different numbers of nodes (variables) and modules (Figure 2C). For the stability profiles, each point represents the ARI at the selected optimal threshold. Note that the optimal thresholding corresponds to the red dashed vertical lines in the simulation stability profiles (Figures 4–6), which is fully detailed later in the Section. For the *p*‐value method, each point represents the performance when the graph is thresholded at the adjusted *p*‐value < 0.05 significance level.

Overall, the performance of the bootstrapped stability is superior to modules detected from graphs derived from *p*‐values. Not surprisingly, the differences in performance are more pronounced for larger *N* (Figure 2A) and in larger networks ((Figure 2B). In terms of the strength of association, note that since *β* is fixed, the difference, *α* − *β*, conveys the strength of the association within a module. We observed that when the absolute difference between *α* and *β* is large (Figure 2A,B), the ARI of our proposed method is stable and close to 1, suggesting that our method gets a perfect module detection. This is because when the absolute difference between *α* and *β* is larger, the associations within modules become larger while the between‐module associations get weaker, thus the modules become easier to identify. A similar trend can be observed when the sample size increases. For example, under *N* = 30, the ARI reaches 1 when *α* = 0.8; if *N* = 50, the ARI attains 1 after *α* = 0.7; if *N* = 100, the ARI is equal to 1 after *α* = 0.6. Figure 2C shows that the *p*‐value approach deteriorates considerably in more complex networks with larger number of modules (Figure 2C). Note that since each point in theses profiles represents the ARI at the optimal threshold, we can also conclude that in more complex networks (Figure 2C), the selection of a smaller threshold than the optimal carries a negligible consequence in terms of stability. Taken together, these results suggest that our method performs better when sample size is large or the within module association is strong. The performance is sustained for more complex networks with large numbers of modules.

**FIGURE 3:**
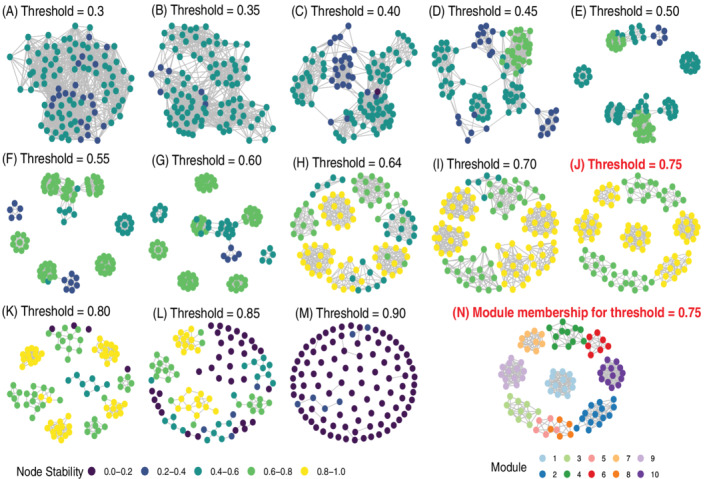
Each threshold *t*_*k*_ in a stability profile represents a graph and subsequent module detection is carried out. Modules are shown for a range of *t*_*k*_ (A–M) corresponding to stability profile for the case with block value 0.8 and *N* = 30 (Figure 4A), where the optimal threshold is selected as *t* = 0.75 (J). In each graph (A–M) the node stability is indicated by color, with yellow representing the most stable nodes (0.8–1.0). The cluster membership for the optimal *t*_*k*_ is shown in (N)

**FIGURE 4:**
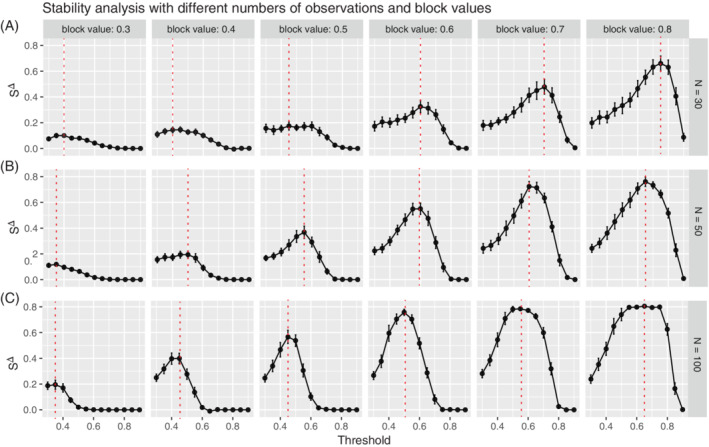
Stability profiles for simulations that capture modules of increasing strength in their association (via correlation block values ∈ [0.30, 0.80]) (columns) are shown (columns), for different sample sizes (rows) (A) *N* = 30, (B) *N* = 50, and (C) *N* = 100. The red dashed line denotes the peak of the profile where the optimal threshold is selected

The stability profile plots are summaries of graph stability across a range of thresholds. Each threshold corresponds to a correlation matrix, graph, and a set of modules. As the threshold increases, the edge density naturally decreases, which measures graph *complexity*. For example, Figure 3A–M shows the node stability on the graphs that correspond to a range of thresholds. This result is from the simulation on a block‐diagonal model, *α* = 0.8, *N* = 30, *p* = 100, modules = 10, and the corresponding stability profile can be seen in the last panel Figure 4A. Not that the peak in the profile (Figure 4A) corresponds to the optimal and most stable threshold, which can be observed in Figure 3J in terms of node stability on the graph, with the corresponding modules are depicted in Figure 3N. Note that the optimal threshold selected by our method is 0.75, which is very close to the true threshold 0.8, with a large group of highly stable nodes. Figure 3N shows a clear selection of 10 modules, which further indicates that our method has the ability to pick up optimal threshold.

Stability profiles were comprehensively examined over a range of simulated block diagonal models. Figure 4 shows a stability profile across a range of threshold values with varying strengths of association (block values) in the modules for different sample sizes. As the sample size increases from *N* = 30 (Figure 4A), and *N* = 50 (Figure 4B), to *N* = 100 (Figure 4C), an increase in stability is observed, which is more pronounced in networks with stronger module associations (e.g., block ∈ {0.60, 0.70, 0.80}). This supports our observations in Figure 2, but now over a range of threshold values that define the stability profile. Comparing across columns in Figure 4, when the difference between *α* and *β* is relatively small, the peak is more pronounced with larger *N*. On the other hand, the need for larger sample size is less of an issue when the difference between *α* and *β* increases. Although we observed a flatted curve for the large sample size (*N* = 100, Figure 4C) and a big difference between *α* and *β*, our method may tend to select a smaller threshold, the consequence is negligible according to Figure 2A,B. Specifically, the change of graph structure with different threshold presented in Figure 3 confirms our assumption that a too‐small threshold leads to a dense and overly complex graph, whereas too large of a threshold results in an overly sparse network, and both cases contains a lot of nodes with the low stability.

**FIGURE 5:**
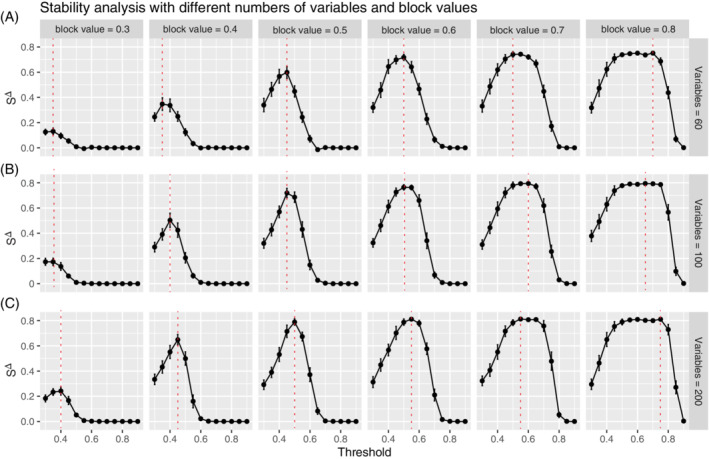
Stability profiles for simulations that capture modules of increasing strength in their association (via correlation block values ∈ [0.30, 0.80]) are shown (columns), for networks of increasing size (rows) (A) *p* = 60, (B) *p* = 100, and (C) *p* = 200. The red dashed line denotes the peak of the profile where the optimal threshold is selected

Stability profiles were also examined for varying strengths of association (block values) in the modules and with different numbers of variables (nodes) (Figure 5). The trends in the stability profiles across block values are similar to those observed in Figure 4. Specifically, Figure 5 demonstrates a more pronounced peak that is observed when there are stronger associations within the modules, which begins to flatten when the associations are at their highest (block = 0.8). On the other hand, Figure 5 shows very subtle differences in the shape of the profile when the number of variables is increased from 60 to 200. However, although the shape is relatively similar, we do observe a systematically higher value of stability as the number of variables increases.

**FIGURE 6:**
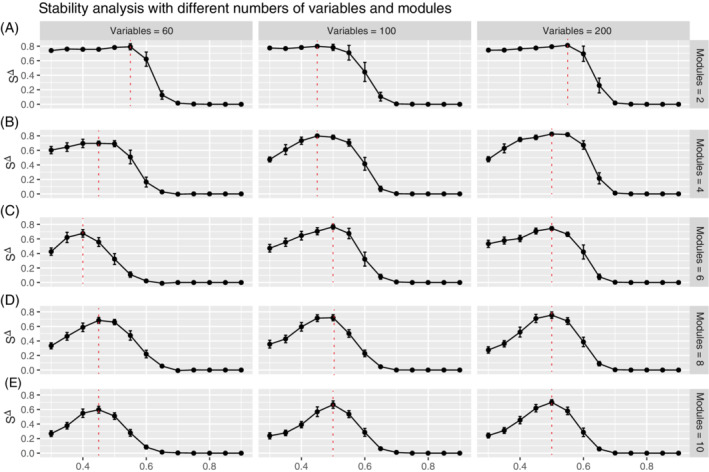
Stability profiles for simulations that capture networks of increasing number of nodes (variables = 60, 100, 200) are shown (columns), for networks that contain different numbers of modules (rows) (A) modules = 2, (B) modules = 4, (C) modules = 6, (D) modules = 8, (E) modules = 10. The red dashed line denotes the peak of the profile where the optimal threshold is selected

Figure 6 shows a similar phenomenon. As the number of variables is varied with the number of modules, we observed no clear difference in the general trend of the profile across the three columns, although the stability value itself slightly higher when the variables are increased from 60 (left column), to 100 (center column), to 200 (right column). This indicates that the changes in the number of variables do not have a major effect on stability estimation. This also suggests that in cases when the module structure is sufficiently complex (Figure 6D,E), larger networks are going to be more stable, as they need to *support* the complex structure. Smaller networks do not support more complex structures (Figure 6D,E left), as the modules are too small and more unstable, regardless of the stability value, the similar profiles, and consequently threshold selection appears to be rather robust to network size. This observation supports what was observed in both Figure 4, and also in Figure 2C, where the *p*‐value method exhibits major sensitivity to graph complexity. Taken together, this phenomenon suggests that the performance of our method is very stable when the graph complexity increases.

**FIGURE 7:**
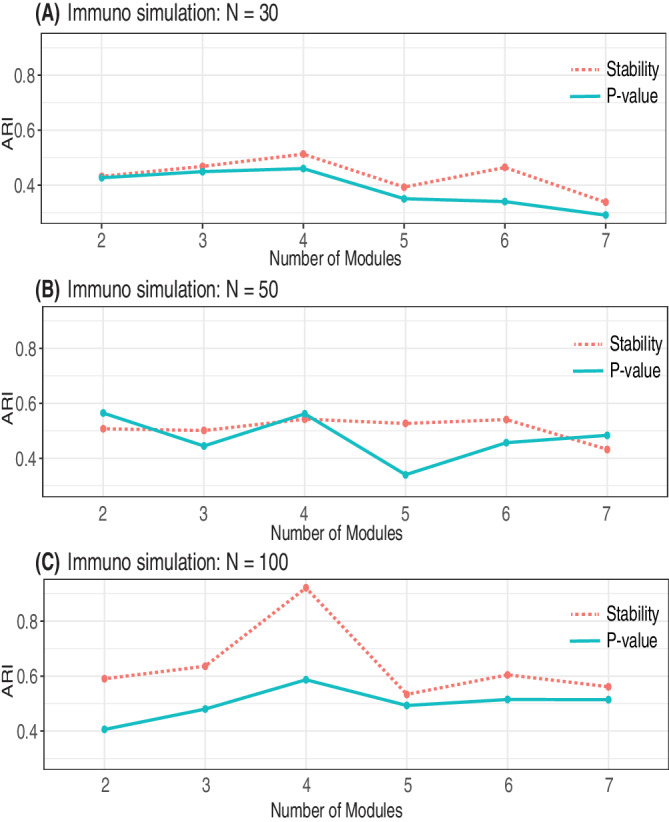
Adjusted rand index (ARI) for simulations based on an immune network in Simulation 2. The bootstrapped stability (red) is compared to a *p*‐value approach (blue) for networks with different numbers of modules and sample size ranged from (A) *N* = 30, (B) *N* = 50, (C) *N* = 100

**FIGURE 8:**
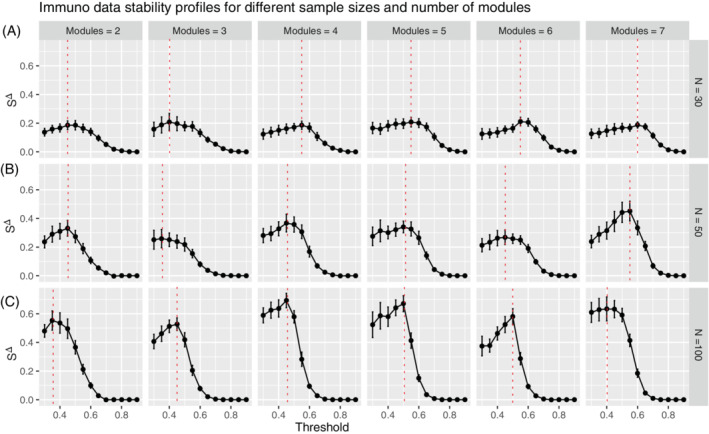
Stability profiles for simulations based on the immuno data are shown for number numbers of modules (modules ∈ {2, 3, 4, 5, 6, 7} (columns) and different sample sizes (rows) (A) *N* = 30, (B) *N* = 50, (C) *N* = 100. The red dashed line denotes the peak of the profile where the optimal threshold is selected

In the immuno data, we observed similar trends (Figure 8) when comparing various sample sizes (*N*), as we observed in our previous block matrix simulations (Figure 4). The peak of a curve is easier to identify with the larger number of observations (*N*), indicating a possible precise estimation of the threshold. However, the increase of the network complexity, which is reflected by the increased number of modules, does not significantly influence the shape of the curve. This observation is in contrast to what was observed in Figure 6. These differences may be due in part to the construction of the immuno simulation, which requires the sequential nesting of modules, and the fact that the more modules we include in the simulation, the more nodes we have in the graph. Due to this fixed relationship between the number of variables and the number of modules, no clear pattern can be observed in Figure 7. However, this phenomenon is not in conflict with what we find in the first simulation example (block diagonal model), if we track the changes of the curve across the diagonal. Take Figure 5C left, Figure 5D middle, and Figure 5E right as an example, we observe that there is no obvious difference between these figures although the number of modules increases from the top left to the bottom right. This is due to the fact that the number of parameters increases at the same time. This is exactly what is observed in the immuno data. Since the relationship between nodes and modules are fixed, increasing modules is actually increasing the number of parameters as well. The ARI is calculated to show the performance of the proposed threshold selection method, and compare it with the *p*‐value based method (Figure 7). A great improvement can be observed along with the increased number of observations for the proposed method. However, no clear difference was observed for different numbers of modules.

**FIGURE 9:**
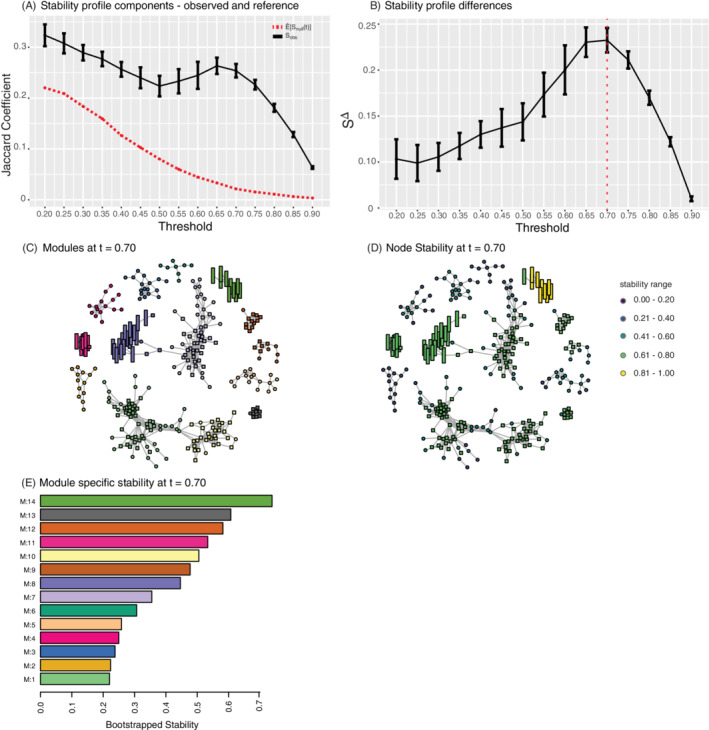
(A) The stability of the observed (black) and expected (red dashed) Jaccard profile. (B) The difference between the observed and expected Jaccard profiles, *S* ^Δ^. (C) Air pollution modules detected using a bootstrap stability estimate of 0.70 resulted in 14 modules. (D) The node stability is indicated with the corresponding color for the threshold *t* = 0.70. (E) The stability of each module is indicated with the corresponding color (indicated in A) for *t* = 0.70

The air pollution metabolomics dataset was also examined using Algorithm 1 over a range of thresholds (Figure 9). The components of the stability profile are shown in Figure 9A,B. Noticeably, the range of stability values is considerably lower, when compared to simulation. This is not surprising in real observational data, which is noisy and has a weaker signal. Note that the signal and trend, in particular, the departure from the stability from the null reference data that has no module structure (Figure 9A,B) is considerable. The difference is at its maximum at approximately 0.70, suggesting this as an optimal graph from the point of view of stability and reproducibility. At this threshold, there are 14 modules detected (Figure 9C). The individual stability of nodes is given in Figure 9D and the individual module stabilities are shown in Figure 9E. At the 0.70 threshold, the final graph and subsequent modules were within our expectations in terms of size and density. Figure 9E shows the nodes stability information with shapes still identified modules. We see that generally, all nodes have relatively high stability, and the nodes in the same modules usually have a similar level of stability. This may also be related to the connectivity patterns.

## 4 DISCUSSION

In this work, we developed an approach to assess the stability of modules detected from a graph. This approach relies on a nonparametric bootstrapping of a dataset to produce a graph based on association, which is then used for module detection. The module detection problem can be viewed as a graph clustering problem. Thus, they share a common challenge, the lack of a gold standard to assess quality and performance, and the lack of methods for the selection of the number of groups (modules/clusters). The notion of stability has been used extensively in the clustering literature as a surrogate for performance [4, 12, 13, 14, 20, 22, 26, 38]. It is therefore natural to bridge stability estimation for clustering to the module detection problem in graphs.

The major contribution of this work is the development of an approach to assess the stability of module detection in a graph at the three levels: (1) level of the node, (2) module, and (3) as a measure for overall performance, and (4) as a means to perform model selection. The latter simultaneously estimates the threshold parameter for defining the graph that produces the most stable and reproducible sets of modules through the comparison of a reference null graph. An important distinction between the work developed in Yu et al. [38] is that we have extended the definitions of stability to a graphical model. Pairwise associations are used to derive a graph, and once identified as significant, these connections are represented with a binary adjacency matrix. Whereas Yu et al. [38] work directly with a clustering problem, where the associations between every pair of variables are used to identify *stable clusters* within a dataset. Notably, alternative definitions of the graph and module detection algorithm can be substituted within this framework. Once the graph is identified, we utilize a scalable fast‐greedy module detection algorithm [6], although there are several alternatives to module detection that could be pursued, see Radicchi et al. [32] for a review. For example, module detection can also be carried out on data that is represented as a sparse weighted graph [33].

Limitations of our approach are shared by clustering problems in general. In our simulations, we demonstrated that the bootstrapping approach to module stability works best when the strength of the association is strong (*α* − *β* is large). In the graph itself, this translates to high levels of connectivity within a module, and low levels of connectivity between modules. Naturally, these well‐defined communities are easily detected and are hardly influenced by the number of variables or the number of observations. On the other hand, when the module structure becomes more obscured, the module detection algorithm will also suffer in terms of performance. In the stability framework, this amounts to more variability in the module detection results across the bootstrap replicates, and consequently lower stability with a higher variance. Taken together, this can make the selection of the optimal threshold using a stability profile challenging. Our approach also works better for larger sample sizes, which naturally give more reliable estimates of correlation. We demonstrated that our method is robust in large graphs with a more complex module structure. However, it is imperative that the graph is rich enough (large enough) to support a more complex module structure. This complexity can arise in terms of the number of communities and the size of the communities.

To the best of authors' knowledge, this is the first application of stability to the area of module detection in a graph. There has been some related work in Bioinformatics, see Li et al. [25] for a survey. However, no approach addresses the same problem of stability estimation at the various levels of the node, module, and for model selection. Beisser et al. [3] developed an approach to identify the accuracy and variability of functional modules. They introduce a concept of consensus modules that are derived from examining nodes and edges across bootstrap replications. They use a predefined network, while our method allows the network structure to change during the resampling, and we are therefore simultaneously estimating the network. The result of this approach is a single module is then investigated to further define sub‐regions, within this module, that have high‐support and functional biological relationships. We view this approach as fundamentally different, as it does not offer the level of granularity for the specifications of several modules, does not offer model selection, and requires a second post hoc level of expertise and interpretation to pull out the sub‐regions that are accurate and functional. Weighted correlation network analysis (WGCNA) [24] is another popular method for identifying modules from a co‐expression network that is represented as a weighted and fully connected undirected graph. The modules are defined as highly interconnected genes (nodes). Within the WGCNA framework, one can identify hub genes, and specify fuzzy memberships, and module significance is defined as the average absolute gene significance measure of genes in a module. Modules from different graphs can be compared for the purpose of understanding module conservation between experimental conditions, for example [19], but not as a means of estimating stability.

The application of the bootstrapping approach to module stability to the metabolomics dataset from an air pollution study [28] demonstrates the utility of this approach for omics data, high‐dimensional data, and network analysis in general. Although the stability levels were in the low range, we were able to determine an appropriate threshold and modules that resulted in biologically meaningful modules. The stability measures directly quantify uncertainty at the metabolite level and the module level enables an investigator to harness additional insights about results and prioritize this information in their conclusions and future hypothesis. Downstream analysis, such as pathway enrichment, can also be strengthened within this framework. For example, stability estimates may be used as node‐level information that can be directly embedded into the enrichment analysis [31]. This will be a direction of future research.

The approach was developed and carried out on correlation graphs. However, this method is generalizable to other graphs that are derived from association measures. In other words, correlation‐based dissimilarity is simply one metric to measure association, and as with most clustering problem, the algorithm lends itself to any measure of dissimilarity that satisfies the metric properties. This approach can also be extended to the structural learning problem in Bayesian networks [23] to both assess the stability of a candidate equivalence class, and when the structure is learned in a sampling framework, the stability estimation can be used for Bayesian model averaging [21]. These extensions will be the subject of future research.

In conclusion, utilizing the notion of *stability* for module detection in a graph enables us to characterize the uncertainty in the modules at the level of the node, module, and overall. Moreover, we demonstrate that this approach can be used to select a threshold parameter from which we define the graph. Our method is suitable for high‐dimensional data and eases the challenge of module interpretation. This approach can be implemented in the bootcluster package in the R programming language.

## Data Availability

Author elects to not share data
